# Effect of Hydroxytyrosol Derivatives of Donepezil on the Activity of Enzymes Involved in Neurodegenerative Diseases and Oxidative Damage

**DOI:** 10.3390/molecules29020548

**Published:** 2024-01-22

**Authors:** Antonio D’Errico, Rosarita Nasso, Rosario Rullo, Jessica Maiuolo, Paola Costanzo, Sonia Bonacci, Manuela Oliverio, Emmanuele De Vendittis, Mariorosario Masullo, Rosaria Arcone

**Affiliations:** 1 Department of Medical, Movement and Well-Being Sciences, University of Naples “Parthenope”, Via Medina, 40, 80133 Napoli, Italy; antonio.derrico002@studenti.uniparthenope.it (A.D.); rosaritanasso@gmail.com (R.N.); rosaria.arcone@uniparthenope.it (R.A.); 2 Institute for the Animal Production Systems in the Mediterranean Environment, Consiglio Nazionale delle Ricerche Piazzale Enrico Fermi 1, 80055 Portici, Italy; rosario.rullo@cnr.it; 3 Department of Health Science, Institute of Research for Food Safety & Health (IRC-FSH), University Magna Græcia of Catanzaro, Viale Europa, 88100 Catanzaro, Italy; maiuolo@unicz.it; 4 Department of Chemistry and Chemical Technologies, University of Calabria, Via P. Bucci, Cubo 12C, 87036 Rende, Italy; paola.costanzo@unical.it; 5 Department of Health Sciences, University Magna Græcia of Catanzaro, Viale Europa, 88100 Catanzaro, Italy; s.bonacci@unicz.it (S.B.); m.oliverio@unicz.it (M.O.); 6 Department of Molecular Medicine and Medical Biotechnology, University of Naples Federico II, Via S. Pansini 5, 80131 Napoli, Italy; devendit@unina.it

**Keywords:** hydroxytyrosol-donepezil hybrid, inflammatory diseases, neurodegenerative disorders, monoamine oxidase (MAO), xanthine oxidase (XO), multitargeting agents

## Abstract

Monoamine oxidase and xanthine oxidase inhibitors represent useful multi-target drugs for the prevention, attenuation, and treatment of oxidative damage and neurodegenerative disorders. Chimeric molecules, constituted by naturally derived compounds linked to drugs, represent lead compounds to be explored for the discovery of new synthetic drugs acting as enzyme inhibitors. We have previously reported that seven hydroxytyrosol-donepezil hybrid compounds play a protective role in an in vitro neuronal cell model of Alzheimer’s disease. In this work, we analyzed the effects exerted by the hybrid compounds on the activity of monoamine oxidase A (MAO-A) and B (MAO-B), as well as on xanthine oxidase (XO), enzymes involved in both neurodegenerative disorders and oxidative stress. The results pointed to the identification, among the compounds tested, of selective inhibitors between the two classes of enzymes. While the 4-hydroxy-3-methoxyphenethyl 1-benzylpiperidine-4-carboxylate- (HT3) and the 4-hydroxyphenethyl 1-benzylpiperidine-4-carboxylate- donepezil derivatives (HT4) represented the best inhibitors of MAO-A, with a scarce effect on MAO-B, they were almost ineffective on XO. On the other hand, the 4,5-dihydroxy-2-nitrophenethyl 1-benzylpiperidine-4-carboxylate donepezil derivative (HT2), the least efficient MAO inhibitor, acted like the best XO inhibitor. Therefore, the differential enzymatic targets identified among the hybrid compounds synthesized enhance the possible applications of these polyphenol-donepezil hybrids in neurodegenerative disorders and oxidative stress.

## 1. Introduction

Inflammatory-driven diseases, including neurodegeneration, have been postulated to include tissue redox state imbalance [1,2,3]. Several enzymatic systems are involved in the regulation of redox metabolisms, which enclose both oxidative stress inducers and their removal [4]. The redox balance is mainly due to the production/elimination of the reactive oxygen species (ROS), namely the superoxide anion radical, the hydroperoxide radical, and hydrogen peroxide. Among the enzymatic activities producing hydrogen peroxide are monoamine oxidases (MAOs) and xanthine oxidases (XOs), which have been used as pharmacological targets to treat specific diseases [5].

MAOs are flavoenzymes located at the outer mitochondrial membrane that exist in two isoenzymatic forms, namely A and B (MAO-A and MAO-B, respectively), that show different tissue-specific expression as well as substrate and inhibitor specificity [6]. MAOs catalyze the oxidative deamination of biogenic amines, including neurotransmitters and drugs, with the production of hydrogen peroxide [7,8]. Although the primary structure of the two enzymes shares 72% sequence identity, differences have been found in the 3D structure of the two enzymes. In particular, the active site of MAO-A is smaller than that of MAO-B, and a different specific conformation of a loop has been reported [9]. Since the two isoenzymes also display different substrate specificity [6,10] and tissue expression [11], they are differentially involved in the regulation of dopamine (MAO-A) or GABA levels (MAO-B) [12]. Therefore, MAO-A and MAO-B have been identified as target enzymes to develop molecules for the treatment of neurodegenerative disorders [7,13,14]. In particular, while inhibitors of MAO-A have been developed to treat depression and anxiety [7,13], those inhibiting MAO-B have been devised to handle Parkinson’s and Alzheimer’s diseases [14]. For these reasons, an increased interest in the design of selective reversible inhibitors for the two isoenzymes has recently developed among researchers [15], even to face the risk of side effects due to the inhibition of MAO-A [16,17].

More recently, the involvement of MAO in cancer and other metabolic diseases such as diabetes, obesity, and cardiovascular diseases has also been reported [18,19,20,21]. In addition, MAO-A inhibitors have been reported to be useful for prostate cancer treatment [22], with a potential dual-action therapy in patients with comorbid depression [23].

XO and xanthine dehydrogenase (XDH) are cytosolic enzymes originating from a common gene transcript, called xanthine oxidoreductase [24,25]. Both enzymes are involved in the catabolism of purines by participating in the homeostasis of several redox species [26]. In physiological conditions, the predominant form is XDH, whereas XO becomes more abundant in oxidative environments. XO catalyzes the conversion of xanthine into uric acid, with the concomitant production of hydrogen peroxide [27]. XO and XDH display a homodimeric structure, and each monomer contains three different domains. In particular, an N-terminal domain containing two iron-sulfur clusters, a middle domain containing a flavine-adenine dinucleotide interacting domain, and a C-terminal domain containing a molybdopterin binding pocket have been identified [28]. From the physiopathological point of view, the upregulation of XO caused by oxidant stress conditions leads to the accumulation of uric acid, a recognized risk factor for gout [29,30]. However, more recently, the upregulation of XO, because of the accumulation of reactive oxygen species, has also been correlated to neurodegenerative diseases [31,32,33,34,35]. In the past, inhibition of XO activity has been chosen as a pharmacological strategy aimed at the reduction of uric acid and hydrogen peroxide production [26,36,37]. This strategy allowed the development of XO inhibitors for the pharmacological treatment of uric acid accumulation diseases [5], with some of them of natural origin [38]. However, it has been reported that the use of these substances can cause undesired side effects that hinder their wider therapeutic use [26,39]. Therefore, the identification of new XO inhibitors with reduced side-adverse effects has recently attracted scientific interest [37,40].

Natural polyphenols possess numerous beneficial properties for human health, including antioxidant, anti-inflammatory, and anticancer properties, among others, slowing the development of several diseases such as cardiovascular, neurodegenerative, and uncontrolled proliferation in cancer [41,42,43,44]. The biological activity of polyphenols is closely related to their antioxidant properties, as they can reduce ROS [45]. To date, the polyphenols of extra virgin olive oil (EVOO), namely secoiridoids and their metabolic derivatives, have been responsible for the recognition of health claims by the EFSA, being particularly protective against various pathologies [46].

**Figure 1:**
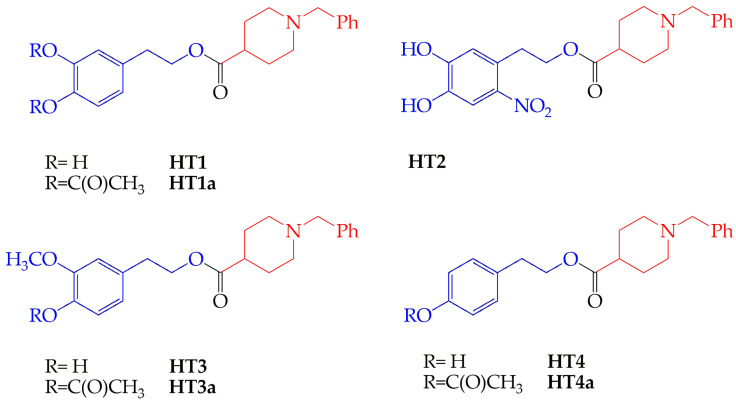
Chemical structures of HT-donepezil hybrids.

In the search for multitargeting agents to treat inflammation-related diseases, there has been a growing interest in pharmacological research that has recently been challenged with the aim of identifying multitargeting molecules useful for the treatment of different diseases [47,48]. In this regard, we have recently proposed that some hybrid compounds, composed of two moieties derived from natural and pharmacological origins, could act as an antioxidant and neuroprotective agents [49,50]. In these compounds, the *N*-benzylpiperidine moiety of donepezil, a drug used to treat Alzheimer’s disease [51], has been linked to different hydroxytyrosol (HT) derivatives (HT1, HT2, HT3, HT4) present in polyphenols of plant origin and then acetylated on the –OH moiety (HT1a, HT3a, HT4a). The acetylation was unstable for one of them (HT2), so the resulting set of free and acetylated HT hybrids was composed of seven new compounds, as represented in Figure 1. They showed interesting antioxidant effects with differentiated mechanisms (hydrogen transfer, electron transfer, and metal chelating mechanisms) depending on the nature of the alcohol moiety [49]. In addition, these molecules exerted a protective action against beta-amyloid-induced cell toxicity, negatively modulating caspase-3 and apoptotic death [50].

In this work, we report the effect of these hydroxytyrosol–donepezil hybrid compounds on the activities of MAO-A and MAO-B, as well as on the XO.

## 2. Results

### 2.1. Effect of HT Hybrids on Monoamine Oxidases Activity

**Figure 2:**
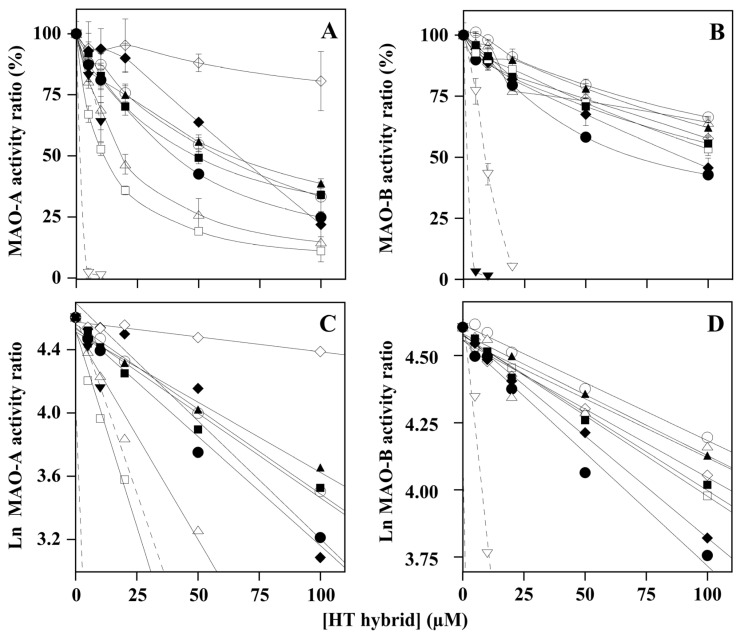
Effect of HT hybrids on monoamine oxidase activity. The activity of MAO-A (**A**) or MAO-B (**B**) was assayed as reported in the Methods section at the indicated concentration of HT1 (○), HT1a (●), HT2 (◊), HT3 (Δ), HT3a (▲), HT4 (□), and HT4a (■) and referred to that measured in the absence of the hybrid compounds. Clorgiline (▽) and selegiline (▼) were used as internal controls of MAO-A and MAO-B inhibition, respectively, and donepezil (♦) was used for comparative purposes. When the same data were analyzed in the semilogarithmic plot (**C**,**D**) for MAO-A and MAO-B, respectively), the squared correlation coefficient *R*^2^ of the straight lines ranged between 0.845 and 0.994.

To test the effect of HT hybrids on MAO activity, the steady-state enzymatic activity of MAO-A or MAO-B was measured as indicated in the Materials and Methods section in the absence or presence of different HT concentrations. The results (Figure 2) indicated that, although with reduced efficacy in comparison with a selective MAO-A (clorgyline) or MAO-B (selegiline) inhibitor, HTs exhibited a dose-dependent inhibition of MAO-A (Figure 2A) and MAO-B (Figure 2B), similar to that exhibited by donepezil. However, a comparison of the various inhibition profiles indicated that all HT hybrids showed a common preference for MAO-A inhibition with respect to MAO-B inhibition. The results were also analyzed through a semilogarithmic transformation of the data (Figure 2C,D), thus allowing the calculation of the concentration for half inhibition values (IC_50_) for all the inhibitors, which were reported in Table 1. Among the hybrid compounds tested in the MAO-A inhibition, the most efficient was HT4 (14.3 µM), followed by HT3 (23.4 µM), whereas the least efficient was HT2 (322 µM); the other HT hybrids showed IC_50_ values ranging in the 44.3–57.0 µM interval. Vice versa, almost no preference towards MAO-B inhibition was observed among the various HT hybrids tested; indeed, all the IC_50_ values ranged in the 85–184 µM interval, with HT2 always endowed with the least efficiency.

The inhibition mechanism of HT hybrids was evaluated through kinetic measurements of MAO activity, as described in the Materials and Methods section. In particular, the initial velocity (*v*_i_) of the reaction was measured at different substrate concentrations in the absence or presence of two fixed inhibitor concentrations. The resulting data were analyzed with both Michaelis–Menten and Lineweaver–Burk plots (see Supplementary Materials for representative experimental results). This procedure allowed an inspection of the inhibition mechanism displayed by the HT hybrids through the effects that each inhibitor exerted on the kinetic parameters *K*_M_ and *V*_max_ of MAO-A or MAO-B activity. The results of this analysis are reported in Table 2 and Table 3 for MAO-A and MAO-B, respectively. Some compounds seemed to act as noncompetitive, uncompetitive, or competitive inhibitors with the corresponding *K*_i_ reported in Table 2 and Table 3, whereas most of them displayed a mixed-type inhibition mechanism. However, as indicated in the Materials and Methods section, an apparent *K*_i_ value could be assigned even to the mixed inhibitors, considering the type of inhibition tentatively approached by the compound based on the experimental data and their graphical representation.

The *K*_i_ values of HT hybrids confirm the previous observation based on the IC_50_ values. Indeed, HT4 (6.1 ± 1.6 µM) and HT3 (7.6 ± 3.7 µM) were the most efficient inhibitors for MAO-A, both possessing an inhibition mechanism approaching competitive, whereas the noncompetitive inhibitor HT2 (398 ± 56 µM) remained the least efficient compound (Table 2). Concerning the MAO-B inhibition, the *K*_i_ values confirm that most of the HT hybrids displayed a lower efficiency compared to MAO-A; furthermore, lower differences exist between the most efficient (HT4a, 20.7 ± 2.8 µM) and least efficient (HT2 and HT3a, 186 ± 98 and 190 ± 37 µM, respectively) compounds in the list (Table 3).

### 2.2. Effect of HT Hybrids on Xanthine Oxidase Activity

**Figure 3:**
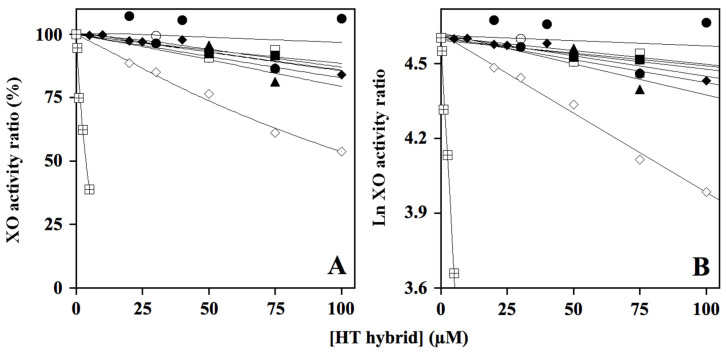
Effect of HT hybrids on xanthine oxidase activity. The steady-state measurements of XO activity (**A**) were realized as reported in the Methods section at the indicated concentration of HT1 (○), HT1a (●), HT2 (◊), HT3 (∆), HT3a (▲), HT4 (□), and HT4a (■) and referred to that measured in the absence of the hybrid compounds. Donepezil (♦) was used for comparative purposes, and allopurinol (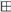) as internal control of XO inhibition. Error standards have not been reported, as they were less than 3% for almost all the measurements. When the same data were analyzed in the semi-logarithmic plot (**B**), the squared correlation coefficient *R*^2^ of the straight lines ranged between 0.761 and 0.997, with the exception of that obtained for compound HT1a, where essentially no inhibition was observed.

Next, we analyzed the effect of HT hybrids on xanthine oxidase activity using an in vitro assay system. To this end, the steady-state enzymatic activity of XO was measured as indicated in the Materials and Methods section, either in the absence or in the presence of different HT concentrations. The results reported in Figure 3 pointed to a clearly evident concentration-dependent inhibition only for one compound, HT2, whereas another compound, HT1a, was almost ineffective in the inhibition of XO activity. An intermediate behavior emerged for the other HT hybrids and donepezil, all of them displaying a similar modest inhibition of the XO activity (Figure 3A). An increase in the HT hybrids’ concentration in Figure 3A was impossible because these compounds became insoluble in the aqueous medium. However, when the data were analyzed through a semilogarithmic transformation (Figure 3B), an IC_50_ value could be extrapolated for all the effective inhibitors and reported in Table 4. As expected, HT2 had the smallest IC_50_ value (130 µM), whereas the other compounds showed values ranging in the 282–605 µM interval, thus confirming their modest inhibition of xanthine oxidase, in comparison with that observed for allopurinol, a well-known XO inhibitor [53].

**Figure 4:**
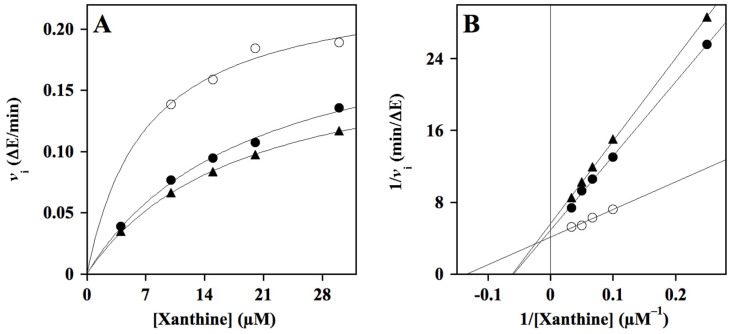
Kinetic analysis of the xanthine oxidase inhibition by HT2. The kinetic measurements of XO activity were realized as reported in the Methods section in the presence of 4–30 µM xanthine concentration, without (○) or with 75 µM (●) or 100 µM (▲) HT2. Data were reported using the hyperbolic Michaelis–Menten equation (**A**) or the Lineweaver–Burk representation (**B**). The correlation coefficients *R*^2^ of the hyperbolic or linear equations ranged between 0.943–0.999 (**A**) and 0.962–0.999 (**B**).

The inhibition power and mechanism of HT2 were also evaluated through kinetic measurements of the XO activity, as indicated in the Material and Methods section. The data analyzed with both the Michaelis–Menten (Figure 4A) and Lineweaver–Burk plots (Figure 4B) suggest that this HT hybrid showed a mixed-type inhibition mechanism with an apparent calculated *K*_i_ value of 63 ± 8 µM, as calculated from an approaching competitive behavior (Table 4).

## 3. Discussion

Chimeric molecules, constituted by naturally derived compounds linked to drugs, represent lead compounds to be explored for the discovery of new synthetic drugs acting as enzyme inhibitors [47,48,54]. We have previously reported that some hydroxytyrosol-donepezil hybrid compounds possess generic antioxidant and metal-chelating activity [49]. In addition, these hydroxytyrosol-donepezil hybrids also elicited a neuroprotective role, as determined in an in vitro model of Alzheimer’s-induced disease in a neuronally differentiated human SH-SY5Y neuroblastoma cell-line [50]. We have also reported that, among the synthesized compounds, some of them possessed predictable pharmacokinetic properties similar to those of donepezil. All these previous findings led to the working hypothesis of considering the hybrid compounds obtained as eligible pharmacophoric/drug candidates [49]. In this study, we have extended the analysis of the effect produced by the hybrid compounds synthesized on the activity of two oxidases involved in inflammatory-driven neurodegenerative and oxidative damage diseases, such as MAOs (A and B isoforms) and XO, respectively. The rational design of the synthesized hybrid molecules was based on the use of two moieties, one derived from donepezil, a known compound for the treatment of Alzheimer’s disease, and the other as hydroxytyrosol derivatives, whose structure is present in natural polyphenols with antioxidant and antiproliferative properties. In the literature, there are different kinds of molecules with multifunctional properties that contain the *N*-benzyl-piperidine moiety associated with MAO and acetylcholinesterase inhibitor activity [55]. Furthermore, the antioxidant properties of phenolic compounds derived from olive oil have been widely demonstrated, which also have beneficial neurochemical effects [56,57]. Considering the similarity between the MAO metabolites and the polyphenolic compounds, it was reasonable that the presence of a phenolic portion could favor one enzyme and also have some effect on others. The results obtained pointed to the identification, among the compounds tested, of selective inhibitors between the two classes of enzymes. In particular, while HT3 and HT4 represent the best inhibitors of MAO-A, with a scarce effect on MAO-B, they were almost ineffective on XO. Furthermore, we also highlighted that the tyrosol moiety of compounds HT3, with only one –OH group on the phenyl ring, is more effective than the other more polar compounds on MAO inhibition. In fact, homovanillyl-derived HT4 with a methoxy group is more effective than the hydroxytyrosol one. On the other hand, HT2, the least efficient inhibitor of MAOs, represents the best inhibitor of XO. In this case, since HT2 is identified as the best metal-chelating compound among the hybrid compounds synthesized [49], an effect on the metal binding site of XO cannot be excluded. In particular, the direct involvement of the HT2 nitro group in the interaction with the iron-sulfur domain of the XO three-dimensional structure can be envisaged. On the other hand, the absence of the nitro group and the metal chelating activity found for HT3 and HT4 [29], almost inactive as XO inhibitors, could support this hypothesis. Furthermore, the presence of the nitro group in HT2 can also be related to the worst effect of this hybrid compound on MAOs, suggesting a possible hindering effect on the interaction of HT2 with the enzyme. The presence of a nitro-catecholic fragment on neuroactive drugs is not a novelty. In fact, both Tolcapone and Entacapone were found to be selective inhibitors of catechol-*O*-methyl transferase (COMT), and it was already demonstrated that the presence of a nitro group on the phenolic ring could enhance the inhibition activity of COMT if compared with hydroxytyrosol [58]. Furthermore, other nitro-compounds were found to be potent inhibitors of xanthine oxidase [39,59]. As expected, the acetylated compounds (HT1a, HT3a, and HT4a) never showed better activity than the not-acetylated ones. Confirming that acetylation, raising the size of the substituent groups, is only a medicinal chemistry tool to enhance membrane permeation [60]. The relevance of the results pointed to a leading approach for the identification of multitargeting hybrid compounds, a strategy receiving increased scientific interest in both neurodegenerative [61,62,63] and other diseases [64], including cancer [65,66].

## 4. Materials and Methods

### 4.1. Materials 

Human monoamine oxidase A and B, bovine milk xanthine oxidase, kynuramine, xanthine, donepezil, clorgyline, selegiline, and allopurinol were purchased from Sigma-Aldrich (Milan, Italy).

HT hybrids were synthesized, purified, and analyzed as previously reported [49]. The set was composed of the following seven new compounds: 3,4-dihydroxyphenetyl 1-benzylpiperidine-4-carboxylate (HT1) and its peracetylated form (HT1a), 4,5-dihydroxy-2-nitrophenethyl 1-benzylpiperidine-4-carboxylate (HT2), 4-hydroxy-3-methoxyphenethyl 1-benzylpiperidine-4-carboxylate (HT3) and its peracetylated form (HT3a), and 4-hydroxyphenethyl 1-benzylpiperidine-4-carboxylate (HT4) and its peracetylated form (HT4a). A 50 mM stock solution of each compound was prepared in DMSO.

### 4.2. Methods

#### 4.2.1. Monoamine Oxidase Assay

Monoamine oxidase activity was assayed by the fluorimetric method, as previously reported [67]. This method, based on the monoamine oxidase oxidation of its non-selective substrate kynuramine, led to the production of 8-hydroxychinoline, which becomes fluorescent in alkaline conditions. In steady-state measurements of MAO activity, the reaction mixture (250 µL), prepared in a 50 mM potassium phosphate buffer, pH 7.1 (buffer A), contained 40 μM kynuramine in the absence or presence of the indicated concentration of the inhibitor. The DMSO concentration carried over by the inhibitor was 0.5% (*v*/*v*), and an identical concentration of DMSO was used in the absence of the inhibitor. The reaction started with the addition of 3.75 μg monoamine oxidase A or B and lasted 20 min. The reaction was stopped by adding 150 μL of 2 M NaOH and, after 10 min of incubation at room temperature, 240 μL of water. After centrifugation (10 min at 15,000 rpm), the fluorescence signal was measured on 500 μL of supernatant using a Cary Eclipse Spectrofluorimeter (Agilent Technologies, Milan, Italy) at room temperature (20–25 °C). Excitation and emission wavelengths of 315 and 380 nm, respectively, were employed; slits were set to 10 nm for both the excitation and emission beams. The residual activity was referred to as that measured in the absence of an inhibitor and expressed as a percentage ratio. The concentration leading to 50% residual activity (IC_50_) was derived from a semilogarithmic plot in which the logarithm of the percentage activity ratio was plotted against inhibitor concentration.

#### 4.2.2. Xanthine Oxidase Assay

Xanthine oxidase activity was assayed through a spectrophotometric method, essentially as previously reported [37,68,69]. In particular, the oxidation of xanthine to uric acid catalyzed by XO was monitored with a Cary 100 UV-Vis Spectrophotometer (Agilent Technologies, Milan, Italy) at 25 °C, thanks to the increase in absorbance at 295 nm due to uric acid formation. In steady-state measurements of XO activity, the assay was carried out in a 500-μL final volume reaction mixture containing 100 mM phosphate buffer, pH 7.8 plus 0.1 mM EDTA (buffer B), 75 μM xanthine, and different concentrations of the various inhibitors. The DMSO concentration carried over by the inhibitor was 1% (*v*/*v*), and an identical concentration of DMSO was used in the absence of the inhibitor. The reaction started with the addition of XO and was followed kinetically for up to 30 s. The initial rate of uric acid formation was expressed as ΔE/min. The effect of each inhibitor in the XO assay was evaluated, and the data were treated as previously indicated for the MAO assay, including the extrapolation of the IC_50_ values.

#### 4.2.3. Kinetics Analysis

The kinetic parameters *K*_M_ and *V*_max_ for MAO and XO substrates were obtained through kinetic measurements of these activities performed at different substrate concentrations, either in the absence or in the presence of two fixed concentrations of the various inhibitors. In particular, the concentration of the substrates kynuramine or xanthine ranged in the 25–150 µM or 4–30 µM interval, respectively. Each assay was realized as reported above, and the values of the initial velocity (*v*_i_) of the reaction were evaluated under the various experimental conditions. The data of *v*_i_ were nonlinearly fitted according to the Michaelis–Menten equation or analyzed by the Lineweaver–Burk equation in order to derive the kinetic parameters in the absence (*K*_M_ and *V*_max_) or in the presence (*K*’_M_ and *V*’_max_) of the inhibitor.

The inhibition power of the various compounds and their inhibition mechanisms were evaluated. Most of them presented a mixed-type inhibition mechanism. However, an apparent inhibition constant (*K*’_i_) was calculated at each inhibitor concentration using the equations hereafter reported, depending on the type of inhibition tentatively approached from the experimental data:

*K*’_i_ = *V*’_max_ × [I]/(*V*_max_ − *V*’_max_) (1)

*K*’_i_ = *K*_M_ × [I]/(*K*’_M_ − *K*_M_) (2)

*K*’_i_ = *K*’_M_ × [I]/(*K*_M_ − *K*’_M_) (3)

If the inhibitor provoked a decrease in the *V*_max_ without apparently changing the *K*_M_, an approaching noncompetitive inhibition was suggested, and Equation (1) was used. If the inhibitor provoked an increase in the *K*_M_ without apparently changing the *V*_max_, an approaching competitive inhibition was suggested, and Equation (2) was used. When both *V*_max_ and *K*_M_ decreased in the presence of the inhibitor, an approaching uncompetitive inhibition was suggested, and Equation (3) or Equation (1) were indifferently used. The *K*’_i_ values obtained at different inhibitor concentrations were then averaged to obtain a unique value of apparent *K*_i_ for each compound.

### 4.3. Statistical Analysis

All data were obtained from at least triplicate experiments and reported as the mean ± standard errors (SE) using a simple weighting method (Student’s *t* test). Graphs were realized using the KaleidaGraph program (Synergy, 5.0 version, Adalta, Italy). The statistical significance of non-linear and linear fittings of the data were evaluated with the squared correlation coefficient *R*^2^.

## 5. Conclusions

In conclusion, the data reported in this paper reinforce the working hypothesis that the identification of hybrid compounds constituted by both known pharmacophoric property moieties and naturally plant-derived portions could represent a leading strategy for the identification of multitargeting agents for the treatment of even unrelated diseases. In addition, the identification of molecules potentially eligible as pharmacophoric/drug candidates with a wider field of applicability can be considered relevant in both reducing undesired side effects of known drugs and/or in treating diseases in comorbidity.

## Figures and Tables

**Table 1:** IC_50_ of HT hybrids for MAO-A and MAO-B inhibition.

Compound	IC_50_ µM ± SD (n)	
	MAO-A	MAO-B
HT1	54.1 ± 6.9 (10)	125 ± 28 (10)
HT1a	50.8 ± 11.0 (10)	85 ± 33 (9)
HT2	322 ± 58 (10)	184 ± 48 (8)
HT3	23.4 ± 6.3 (10)	171 ± 44 (10)
HT3a	44.3 ± 14.4 (10)	174 ± 80 (10)
HT4	14.3 ± 2.2 (8)	106 ± 22 (6)
HT4a	57.0 ± 18.2 (10)	86 ± 24 (6)
Donepezil	67.6 ± 8.1 (10)	40.0 ± 10.9 (10)
Clorgyline ^1^	0.15 ± 0.02 (3)	4.3 ± 0.1 (3)
Seligiline ^2^	15.9 ± 0.1 (3)	0.23 ± 0.05 (3)

^1^ MAO-A control inhibition. ^2^ MAO-B control inhibition.

**Table 2:** Kinetic analysis of the HT hybrids for MAO-A inhibition.

Compound	Inhibition Type	Note	*K*_i_, Calculated from	
			*K*_M_ Increase	*V*_max_ Decrease
			µM ± SE (n)	
HT1	mixed	approaching competitive	39.7 ± 14.3 (8)	
HT1a	mixed	approaching competitive	36.5 ± 13.2 (8)	
HT2	noncompetitive	-		398 ± 56 (8)
HT3	mixed	approaching competitive	7.6 ± 3.7 (8)	
HT3a	uncompetitive	-		80.5 ± 30.4 (13) ^a^
HT4	mixed	approaching competitive	6.1 ± 1.6 (8)	
HT4a	mixed	approaching competitive	15.6 ± 4.2 (8)	
Donepezil	mixed	approaching noncompetitive		82.9 ± 10.0 (8)
Clorgyline ^b^	Inactivator [52]	-		0.012 [52]

^a^ average value from *V*_max_ or *K*_M_ decrease. ^b^ control of MAO-A inhibition.

**Table 3:** Kinetic analysis of the HT hybrids for MAO-B inhibition.

Compound	Inhibition Type	Note	*K*_i_, Calculated from	
			*K*_M_ Increase	*V*_max_ Decrease
			µM ± SE (n)	
HT1	competitive	-	163 ± 28 (8)	
HT1a	mixed	approaching competitive	37.9 ± 13.1 (6)	
HT2	uncompetitive	-		186 ± 98 (12) ^a^
HT3	mixed	approaching competitive	115 ± 50 (6)	
HT3a	mixed	approaching competitive	190 ± 37 (4)	
HT4	uncompetitive	-		128 ± 45 (14) ^a^
HT4a	competitive	-	20.7 ± 2.8 (8)	
Donepezil	mixed	approaching competitive	15.3 ± 4.6 (6)	
Selegiline ^b^	Inactivator [52]			0.055 [52]

^a^ average value from *V*_max_ or *K*_M_ decrease. ^b^ control of MAO-B inhibition.

**Table 4:** IC_50_ of HT hybrids for XO inhibition and mechanism of inhibition for the compound HT2.

Compound	IC_50_	Inhibition Type	Note	*K*_i_
				Calculated from *K*_M_ Increase
	µM ± SE (n)			µM ± SE (n)
HT1	454 ± 22 (2)			
HT1a	no inhibition			
HT2	109 ± 20 (4)	mixed	approaching competitive	63 ± 8 (4)
HT3	361 ± 1 (2)			
HT3a	282 ± 37 (2)			
HT4	605 ± 35 (2)			
HT4a	438 ± 6 (2)			
Donepezil	424 ± 23 (2)			
Allopurinol	3.7 ± 0.5 (2)	Competitive [53]	-	1.8 [53]

## Data Availability

Data are contained within the article and supplementary materials.

## Supplementary Materials

The following supporting information can be downloaded at: https://www.mdpi.com/article/10.3390/molecules29020548/s1, Supplementary Figures S1–S8: Representative experiments for the determination of the kinetic parameters of MAO-A or MAO-B activity.
