# Supplementary material for: Effect of Hydroxytyrosol Derivatives of Donepezil on the Activity of Enzymes Involved in Neurodegenerative Diseases and Oxidative Damage
Source: Molecules. 2024 Jan 22;29(2):548. doi: 10.3390/molecules29020548 (PMC10819651; doi:10.3390/molecules29020548)

Supplementary material

Effect of hydroxytyrosol derivatives of Donepezil on the activity of enzymes involved in neurodegenerative diseases and oxidative damage.

Antonio D'Errico[1*], Rosarita Nasso[1*], Rosario Rullo[2*], Jessica Maiuolo[3], Paola Costanzo[4], Sonia Bonacci[5], Manuela Oliverio[5], Emmanuele De Vendittis[6], Mariorosario Masullo[1], Rosaria Arcone[1]

Affiliations

[1] Department of Medical, Movement and Well-Being Sciences, University of Naples "Parthenope", Via Medina, 40, 80133 Napoli, Italy.
[2] Institute for the Animal Production Systems in the Mediterranean Environment, CNR, Piazzale Enrico Fermi 1, 80055 Portici, Italy.
[3] Department of Health Science, Institute of Research for Food Safety & Health (IRC-FSH), University Magna Græcia of Catanzaro, Viale Europa, 88100 Catanzaro, Italy.
[4] Department of Chemistry and Chemical Technologies, University of Calabria, Via P. Bucci, Cubo 12C, 87036 Rende, Italy.
[5] Department of Health Sciences, University Magna Græcia of Catanzaro, Viale Europa, 88100 Catanzaro, Italy.
[6] Department of Molecular Medicine and Medical Biotechnology, University of Naples Federico II, Via S. Pansini 5, I-80131 Napoli, Italy.
*Equally contributed to this work

Supplementary Figures S1 – S8: Representative experiments for the determination of the kinetic parameters of MAO-A or MAO-B activity.

Representative experiments for the determination of the kinetic parameters of MAO-A or MAO-B activity in the absence or in the presence of the indicated concentration of Donepezyl (Figure S1), HT1 (Figure S2), HT1a (Figure S3), HT2 (Figure S4), HT3 (Figure S5), HT3a (Figure S6), HT4 (Figure S7) or HT4a (Figure S8). The data were interpolated by non-linear fitting in the hyperbolic Michaelis Menten equation (panels A) or by linear fitting in the Lineweaver-Burk equation (panels B) using the Kaleidagraph application (Synergy) version 5.0.4 for Apple MacOS. Fitting parameters furnished by the application were reported at the bottom of each graph.

Figure S1_A. Effect of Donepezyl on the kinetics data of MAO-A activity.

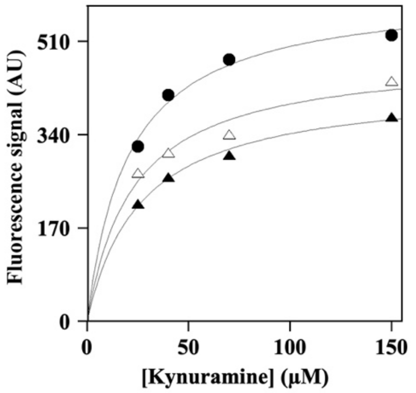

MAO-A alone (filled circles) y = m1*x/(m2+x)		
	Value	Error
m1	602.26	25.495
m2	20.245	3.2025
Chisq	521.3	NA
R^2	0.97762	

+ 20 μM Donepezil (empty triangles) y = m1*x/(m2+x)		
	Value	Error
m1	485.16	40.254
m2	22.509	6.5875
Chisq	1191.7	NA
R^2	0.92324	

+ 40 μM Donepezil (filled triangles) y = m1*x/(m2+x)		
	Value	Error
m1	431.39	16.034
m2	26.233	3.1761
Chisq	164.78	NA
R^2	0.9878	

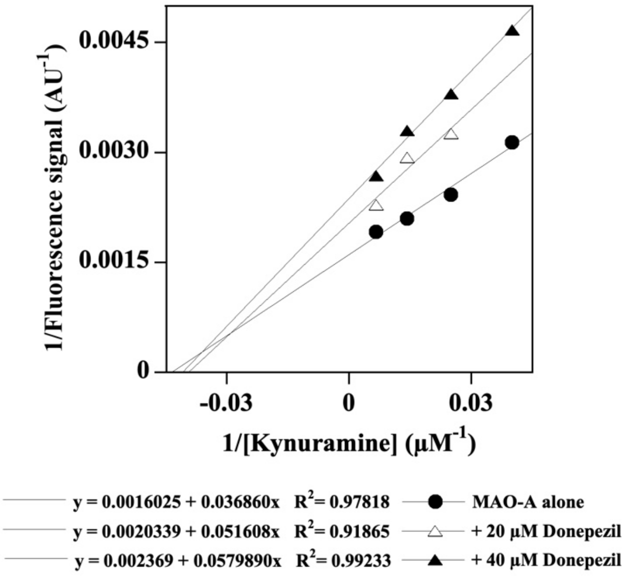

Figure S1_B. Effect of Donepezyl on the kinetics data of MAO-B activity.

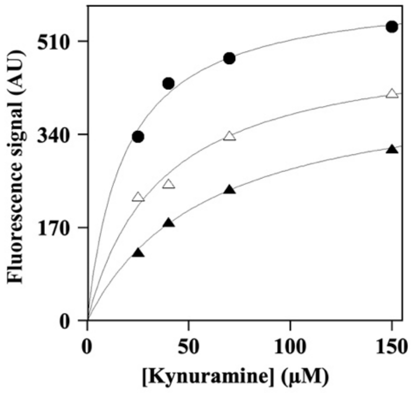

MAO-B alone (filled circles) y = m1*x/(m2+x)		
	Value	Error
m1	606.57	24.495
m2	18.407	2.931
Chisq	517.23	NA
R^2	0.97595	

+ 20 μM Donepezil (empty triangles) y = m1*x/(m2+x)		
	Value	Error
m1	511.55	37.58
m2	35.991	7.4059
Chisq	647.46	NA
R^2	0.97124	

+ 40 μM Donepezil (filled triangles) y = m1*x/(m2+x)		
	Value	Error
m1	440.21	13.933
m2	59.288	4.2701
Chisq	44.529	NA
R^2	0.99774	

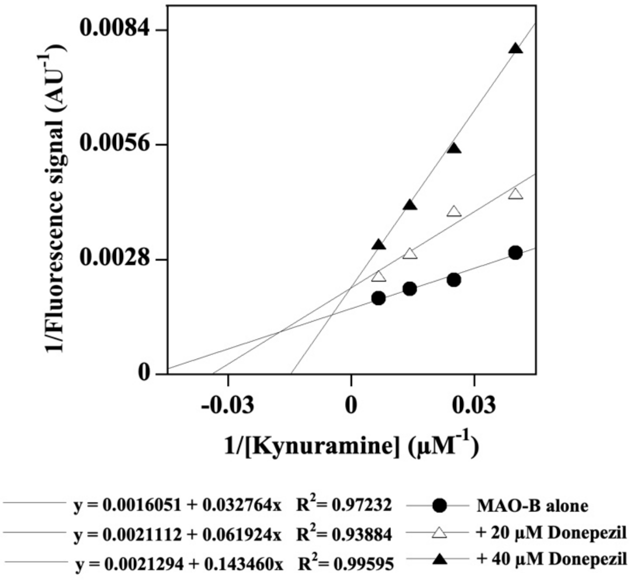

Figure S2_A. Effect of HT1 on the kinetics data of MAO-A activity.

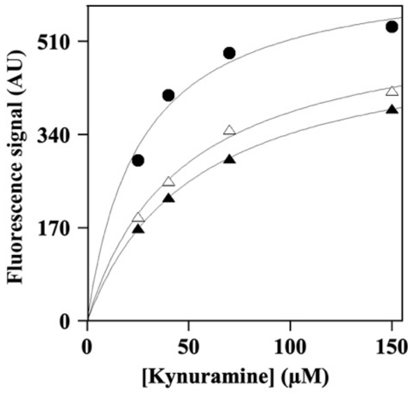

MAO-A alone (filled circles) y = m1*x/(m2+x)		
	Value	Error
m1	645.86	47.716
m2	25.798	6.2615
Chisq	1482.6	NA
R^2	0.95639	

+ 20 μM HT1 (empty triangles) y = m1*x/(m2+x)		
	Value	Error
m1	556.9	27.833
m2	46.166	5.8029
Chisq	258.34	NA
R^2	0.99163	

+ 40 μM HT1 (filled triangles) y = m1*x/(m2+x)		
	Value	Error
m1	522.26	4.1837
m2	52.825	1.0075
Chisq	4.8052	NA
R^2	0.99982	

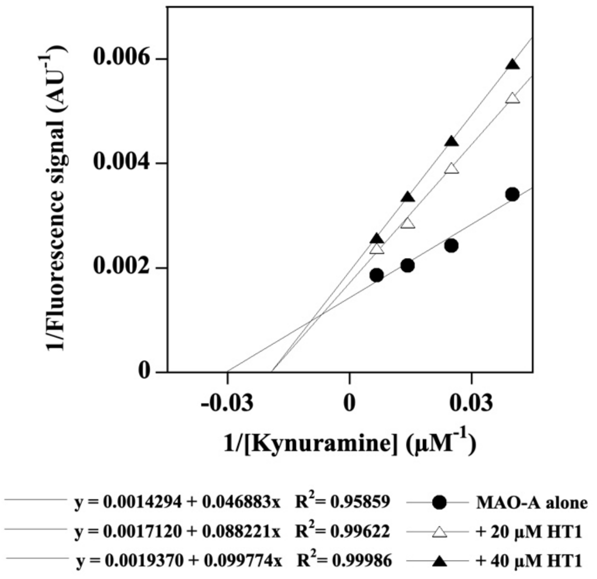

Figure S2_B. Effect of HT1 on the kinetics data of MAO-B activity.

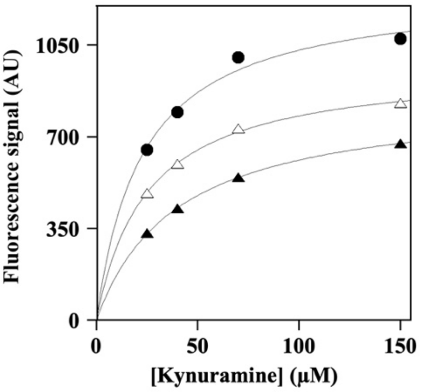

MAO-B alone (filled triangles) y = m1*x/(m2+x)		
	Value	Error
m1	1264.7	65.59
m2	22.727	4.1361
Chisq	3138	NA
R^2	0.97211	

+ 25 μM HT1 (empty triangles) y = m1*x/(m2+x)		
	Value	Error
m1	973.03	19.81
m2	24.83	1.6936
Chisq	264.74	NA
R^2	0.99611	

+ 50 μM HT1 (filled triangles) y = m1*x/(m2+x)		
	Value	Error
m1	852.44	3.8973
m2	39.509	0.48544
Chisq	6.2178	NA
R^2	0.99991	

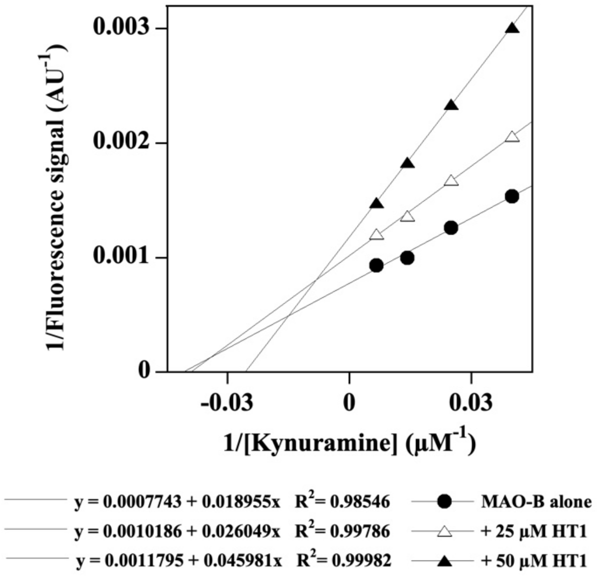

Figure S3_A. Effect of HT1a on the kinetics data of MAO-A activity.

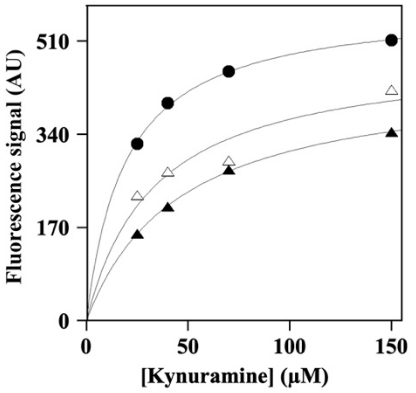

MAO-A alone (filled circles) y = m1*x/(m2+x)		
	Value	Error
m1	578.63	8.2861
m2	19.183	1.058
Chisq	57.4	NA
R^2	0.99706	

+ 20 µM HT1a (empty triangles) y = m1*x/(m2+x)		
	Value	Error
m1	490.59	69.152
m2	33.465	13.66
Chisq	2383.7	NA
R^2	0.88453	

+ 40 µM HT1a (filled triangles) y = m1*x/(m2+x)		
	Value	Error
m1	450.65	6.568
m2	45.808	1.6845
Chisq	14.541	NA
R^2	0.99925	

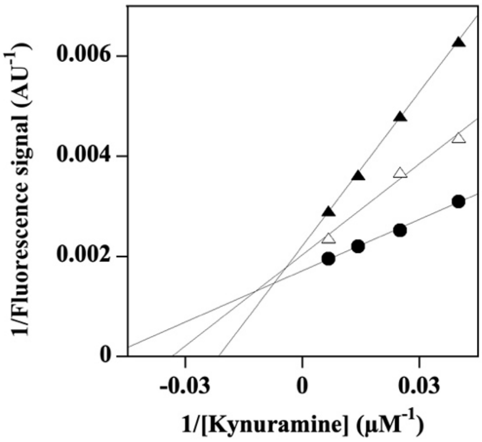

y = 0.0017108 + 0.03410x R^2= 0.99626 — MAO-A alone

y = 0.0020313 + 0.06049x R^2= 0.98662 — + 20 µM HT1a

y = 0.0022085 + 0.10239x R^2= 0.99949 — + 40 µM HT1a

Figure S3_B. Effect of HT1a on the kinetics data of MAO-B activity.

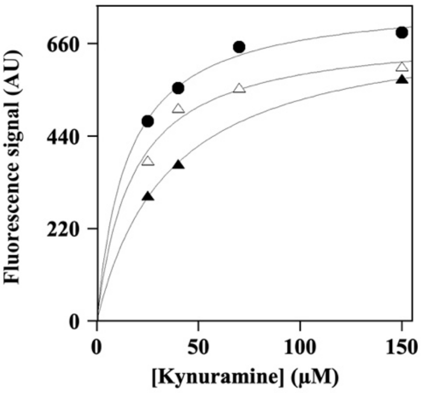

MAO-B alone (filled circles) y = m1*x/(m2+x)		
	Value	Error
m1	766.91	23.617
m2	14.916	2.053
Chisq	554.35	NA
R^2	0.97979	

+ 20 µM HT1a (empty triangles) y = m1*x/(m2+x)		
	Value	Error
m1	688.29	40.118
m2	17.486	4.1402
Chisq	1439.6	NA
R^2	0.94807	

+ 40 µM HT1a (empty triangles) y = m1*x/(m2+x)		
	Value	Error
m1	712.89	12.62
m2	35.526	1.7376
Chisq	35.576	NA
R^2	0.99915	

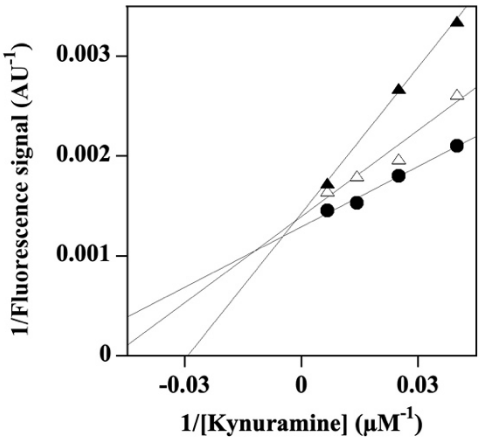

y = 0.0012919 + 0.020121x R^2= 0.98780 — MAO-B alone

y = 0.0013937 + 0.028728x R^2= 0.94653 — + 20 µM HT1a

y = 0.0014250 + 0.048672x R^2= 0.99837 — + 40 µM HT1a

Figure S4_A. Effect of HT2 on the kinetics data of MAO-A activity.

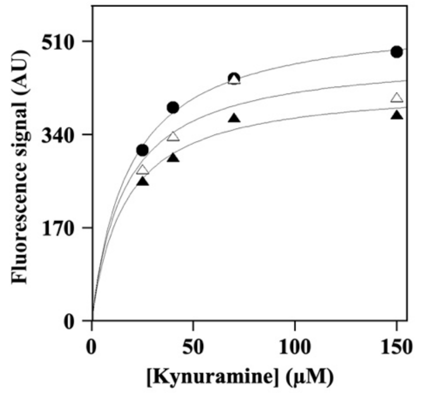

MAO-A alone (filled circles) y = m1*x/(m2+x)		
	Value	Error
m1	555.47	14.29
m2	18.427	1.8681
Chisq	175.89	NA
R^2	0.99004	

+ 50 μM HT2 (empty triangles) y = m1*x/(m2+x)		
	Value	Error
m1	484.82	61.206
m2	16.446	8.7455
Chisq	3495.1	NA
R^2	0.78448	

+ 100 μM HT2 (filled triangles) y = m1*x/(m2+x)		
	Value	Error
m1	431	27.286
m2	16.437	4.3846
Chisq	694.87	NA
R^2	0.93211	

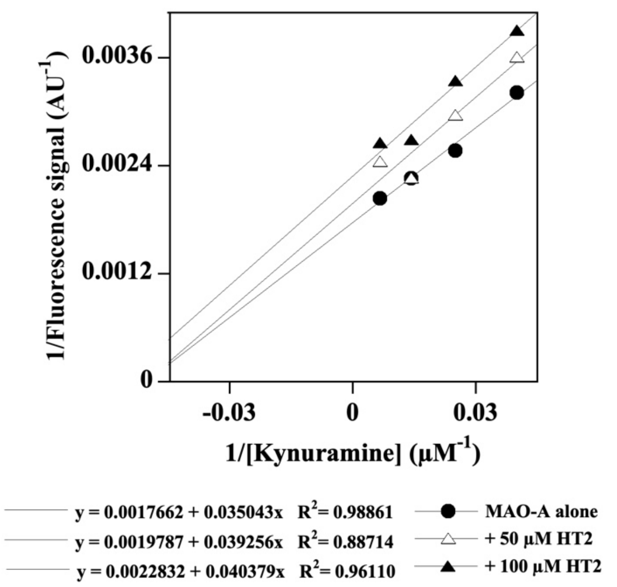

Figure S4_B. Effect of HT2 on the kinetics data of MAO-B activity.

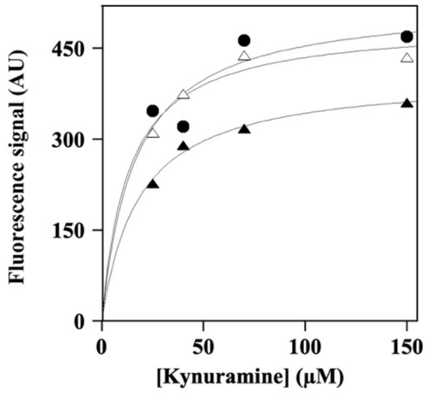

MAO-B alone (filled circles) y = m1*x/(m2+x)		
	Value	Error
m1	527.31	72.013
m2	16.239	9.4125
Chisq	4879.6	NA
R^2	0.72648	

+ 50 μM HT2 (empty triangles) y = m1*x/(m2+x)		
	Value	Error
m1	491.59	30.984
m2	13.095	4.0049
Chisq	1030.7	NA
R^2	0.90579	

+ 100 μM HT2 (filled triangles) y = m1*x/(m2+x)		
	Value	Error
m1	404.82	15.144
m2	18.152	2.6992
Chisq	199.72	NA
R^2	0.97861	

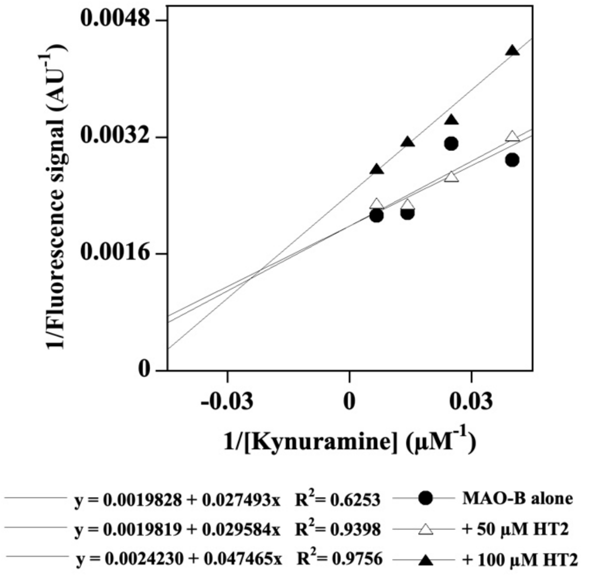

Figure S5_A. Effect of HT3 on the kinetics data of MAO-A activity.

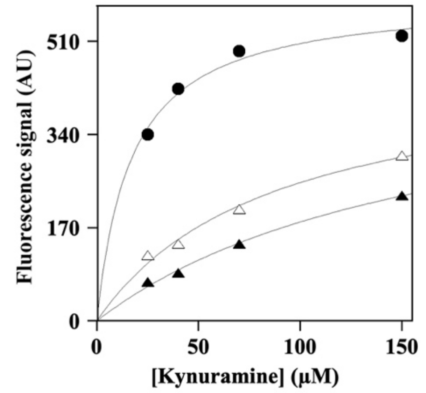

MAO-A alone (filled circles) $y = m1*x/(m2+x)$	Value	Error
m1	592.15	25.666
m2	16.997	3.043
Chisq	600.97	NA
R^2	0.96893	

+ 20 µM HT3 (empty triangles) $y = m1*x/(m2+x)$	Value	Error
m1	471.97	52.83
m2	86.986	19.248
Chisq	321.05	NA
R^2	0.98402	

+ 40 µM HT3 (filled triangles) $y = m1*x/(m2+x)$	Value	Error
m1	484.61	65.859
m2	168.56	36.584
Chisq	106.55	NA
R^2	0.99297	

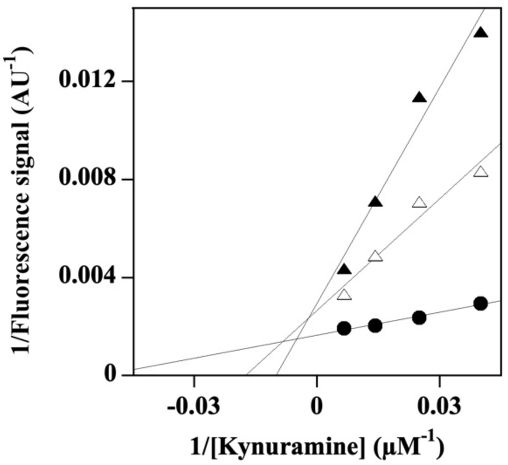

$y = 0.0016418 + 0.03131x$ $R^2 = 0.97654$ — MAO-A alone

$y = 0.0026531 + 0.15179x$ $R^2 = 0.95406$ — + 20 µM HT3

$y = 0.0029276 + 0.29339x$ $R^2 = 0.96524$ — + 40 µM HT3

Figure S5_B. Effect of HT3 on the kinetics data of MAO-B activity.

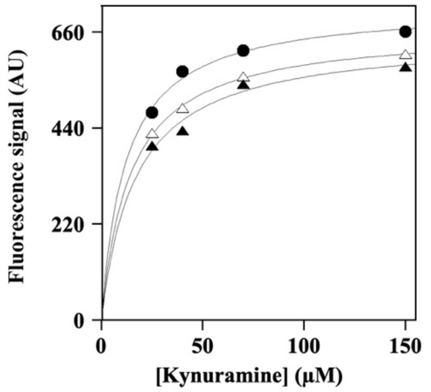

MAO-B alone (filled circles) $y = m1*x/(m2+x)$	Value	Error
m1	720.27	19.497
m2	11.991	1.6682
Chisq	428.1	NA
R^2	0.97752	

+ 20 µM HT3 (empty triangles) $y = m1*x/(m2+x)$	Value	Error
m1	667.49	8.2996
m2	14.281	0.81546
Chisq	70.312	NA
R^2	0.9963	

+ 40 µM HT3 (filled triangles) $y = m1*x/(m2+x)$	Value	Error
m1	649.5	31.553
m2	16.763	3.3914
Chisq	916.96	NA
R^2	0.95821	

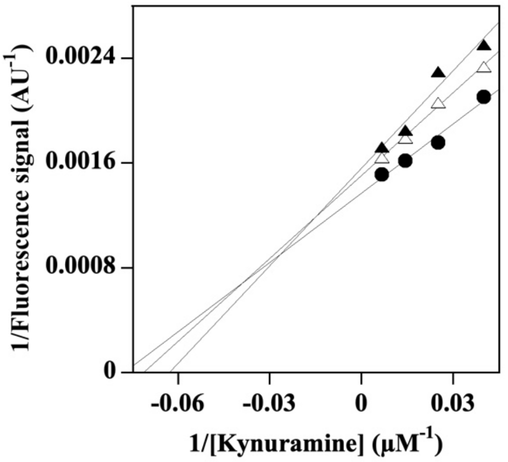

$y = 0.0013700 + 0.017628x$ $R^2 = 0.97655$ — MAO-B alone

$y = 0.0015039 + 0.021103x$ $R^2 = 0.99562$ — + 20 µM HT3

$y = 0.0015607 + 0.024829x$ $R^2 = 0.94913$ — + 40 µM HT3

Figure S6_A. Effect of HT3a on the kinetics data of MAO-A activity.

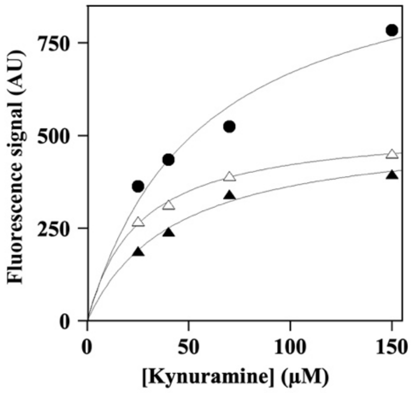

MAO-A alone (filled circles) y = m1*x/(m2+x)		
	Value	Error
m1	1041.3	144.9
m2	55.722	18.075
Chisq	5312.7	NA
R^2	0.94757	

+ 20 μM HT3a (empty triangles) y = m1*x/(m2+x)		
	Value	Error
m1	527.41	14.142
m2	25.317	2.2517
Chisq	132.53	NA
R^2	0.99332	

+ 40 μM HT3a (filled triangles) y = m1*x/(m2+x)		
	Value	Error
m1	518.8	38.615
m2	42.884	8.2803
Chisq	549.37	NA
R^2	0.97938	

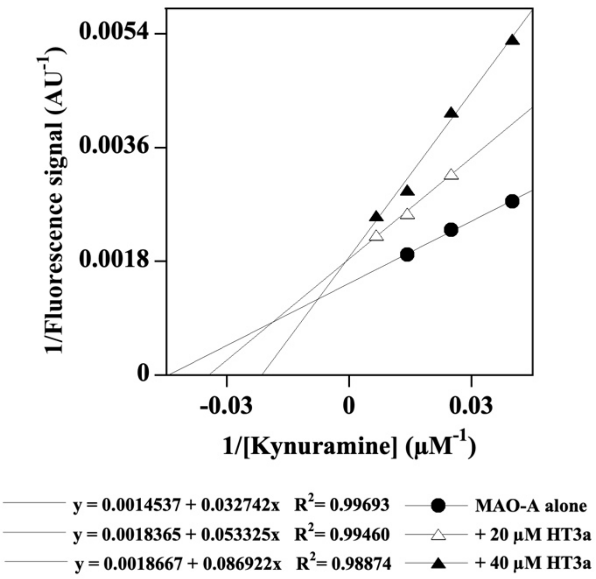

Figure S6_B. Effect of HT3a on the kinetics data of MAO-B activity.

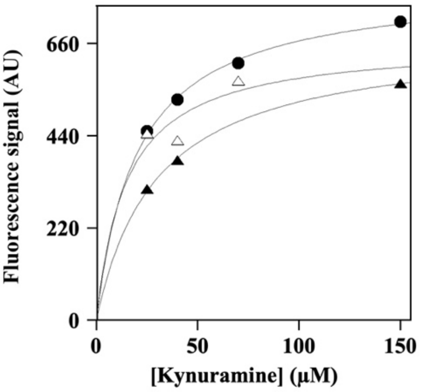

MAO-B alone (filled circles) y = m1*x/(m2+x)		
	Value	Error
m1	801.47	15.695
m2	20.309	1.4836
Chisq	197.08	NA
R^2	0.99484	

+ 50 μM HT3a (empty triangles) y = m1*x/(m2+x)		
	Value	Error
m1	663.47	177.66
m2	14.974	14.939
Chisq	4362.4	NA
R^2	0.63778	

+ 100 μM HT3a (filled triangles) y = m1*x/(m2+x)		
	Value	Error
m1	676.63	13.296
m2	29.919	1.7387
Chisq	46.66	NA
R^2	0.99863	

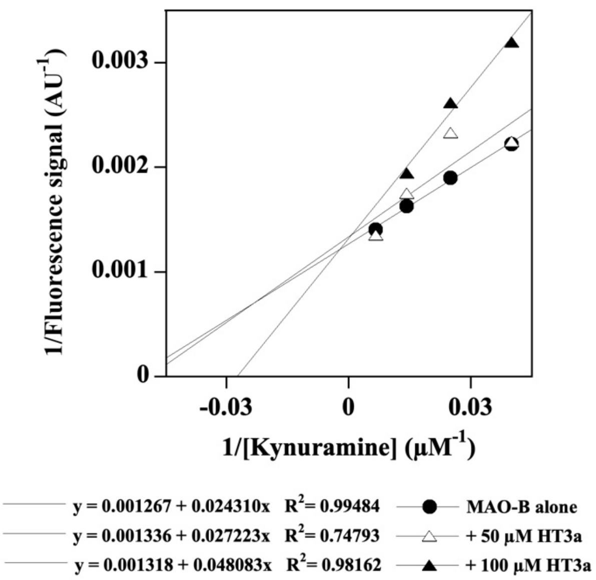

Figure S7_A. Effect of HT4 on the kinetics data of MAO-A activity.

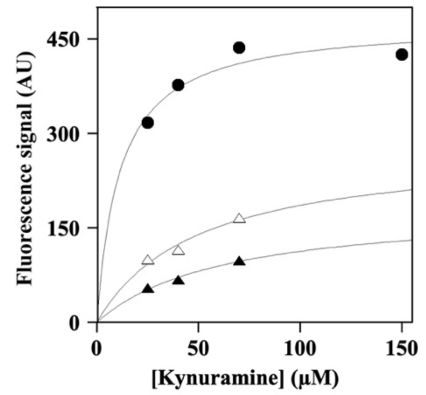

MAO-A alone (filled circles) y = m1*x/(m2+x)		
	Value	Error
m1	476.66	31.411
m2	11.236	3.9748
Chisq	1148.7	NA
R^2	0.87129	

+ 20 μM HT4 (empty triangles) y = m1*x/(m2+x)		
	Value	Error
m1	277.51	73.701
m2	49.532	25.854
Chisq	128.42	NA
R^2	0.94589	

+ 40 μM HT4 (filled triangles) y = m1*x/(m2+x)		
	Value	Error
m1	183.47	36.323
m2	62.835	22.21
Chisq	18.572	NA
R^2	0.98079	

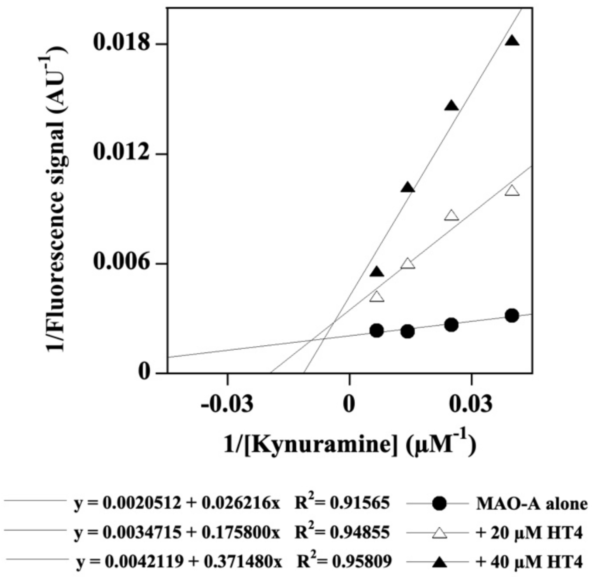

Figure S7_B. Effect of HT4 on the kinetics data of MAO-B activity.

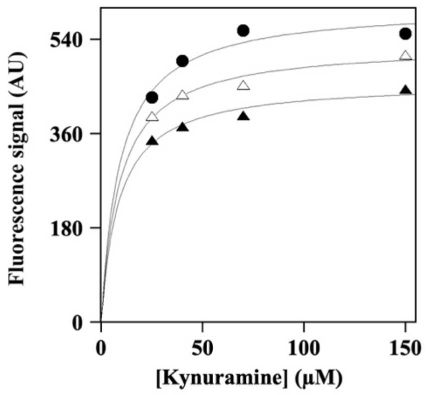

MAO-B alone (filled circles) y = m1*x/(m2+x)		
	Value	Error
m1	603.39	28.874
m2	9.145	2.7112
Chisq	1066.6	NA
R^2	0.89836	

+ 20 μM HT4 (empty triangles) y = m1*x/(m2+x)		
	Value	Error
m1	529.68	17.793
m2	8.9926	1.8941
Chisq	407.86	NA
R^2	0.9421	

+ 40 μM HT4 (filled triangles) y = m1*x/(m2+x)		
	Value	Error
m1	458.8	16.837
m2	8.6613	2.0479
Chisq	370.85	NA
R^2	0.92648	

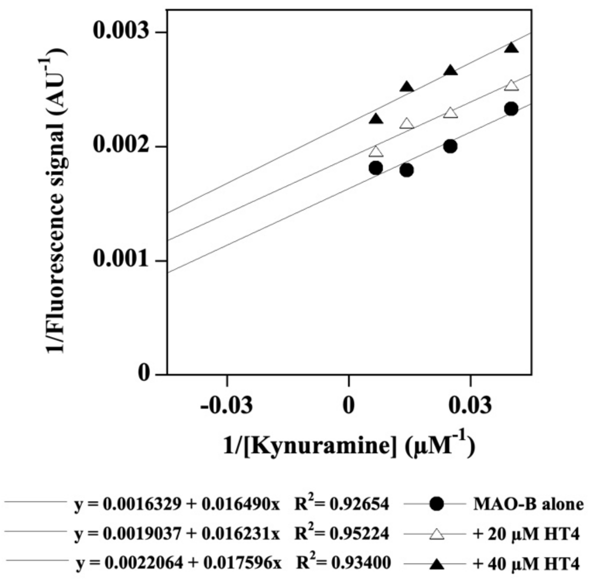

Figure S8_A. Effect of HT4a on the kinetics data of MAO-A activity.

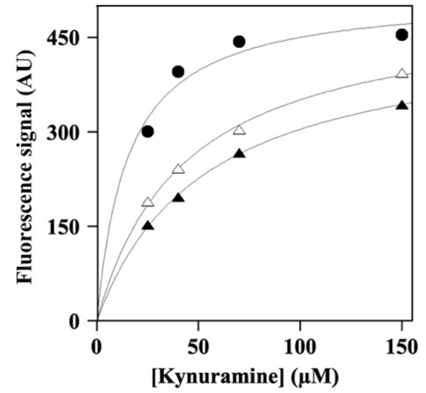

MAO-A alone (empty triangles) y = m1*x/(m2+x)		
	Value	Error
m1	518.45	38.501
m2	15.234	4.9913
Chisq	1453.9	NA
R^2	0.90124	

+ 20 μM HT4a (empty triangles) y = m1*x/(m2+x)		
	Value	Error
m1	501.73	15.911
m2	42.991	3.533
Chisq	92.967	NA
R^2	0.99598	

+ 40 μM HT4a (filled triangles) y = m1*x/(m2+x)		
	Value	Error
m1	463.62	9.961
m2	52.484	2.6916
Chisq	27.507	NA
R^2	0.99869	

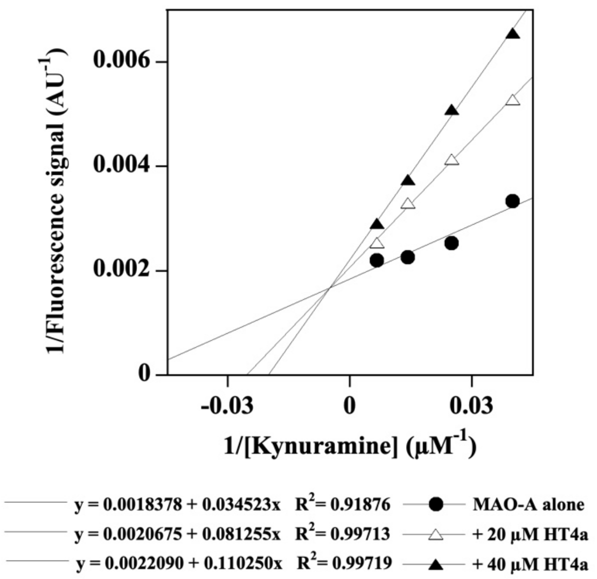

Figure S8_B. Effect of HT4a on the kinetics data of MAO-B activity.

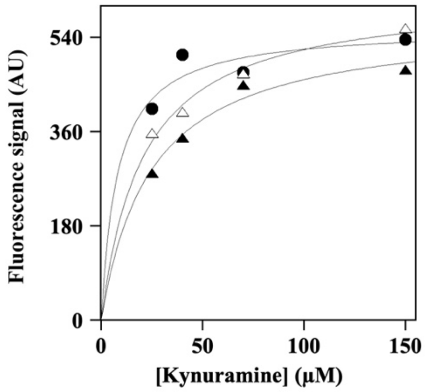

MAO-B alone (filled circles) y = m1*x/(m2+x)		
	Value	Error
m1	558.83	46.587
m2	8.0907	4.5679
Chisq	2915.4	NA
R^2	0.69848	

+ 20 μM HT4a (empty triangles) y = m1*x/(m2+x)		
	Value	Error
m1	623.01	28.3
m2	20.62	3.4648
Chisq	633.06	NA
R^2	0.97283	

+ 40 μM HT4a (filled triangles) y = m1*x/(m2+x)		
	Value	Error
m1	571.6	37.102
m2	24.529	5.3676
Chisq	938.94	NA
R^2	0.96225	

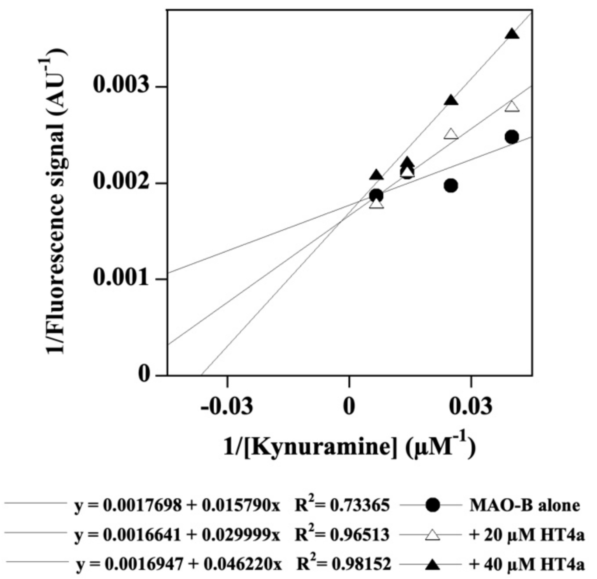

Supplement: Supplementary file 1 [file molecules-29-00548-s001.zip › molecules-2827776-supplementary.pdf]
